# Perforation gastrique idiopathique du nouveau-né: à propos d’un cas

**DOI:** 10.11604/pamj.2017.27.136.12036

**Published:** 2017-06-22

**Authors:** Fatima Zahra Aglili, Maha Oudrhiri, Houria Knouni, Younes Taboz, Hasna Benkiran, Hassan Aguenaou, Fouad Ettaybi, Amina Barkat

**Affiliations:** 1 Service de Médecine et Réanimation Néonatale, PV, HER, Chis Ibn Sina, Commission de Formation Médicale Continue Université Mohammed V, Faculté de Médecine et Pharmacie, Équipe de Recherche en Santé et Nutrition du Couple Mère Enfant; 2 Unité Mixte de Recherche en Nutrition et Alimentation URAC 39, Université Ibn Tofail-CNESTEN, RDC-Nutrition AFRA/AIEA, Morrocco; 3 Service des Urgences Chirurgicales de l’Hôpital d’Enfant Rabat

**Keywords:** Nouveau-né, détresse abdominale, abdomen sans préparation, perforation gastrique spontanée, Newborn, abdominal distress, abdomen without prior treatment, spontaneous gastric perforation

## Abstract

La perforation gastrique néonatale spontanée est rare. Nous rapportons un cas survenu chez un nouveau-né issu d'une grossesse et une naissance sans anomalies, et qui a présenté au troisième jour de sa vie brutalement une distension abdominale importante, suivie des vomissements bilieux. La radiographie de l'abdomen sans préparation montrait un pneumopéritoine massif, la laparotomie trouvait une perforation au niveau de la paroi gastrique antérieure, qui était suturée en un plan. Les suites opératoires étaient simples. L'évolution des perforations gastriques spontanées survenant chez le nouveau-né est habituellement favorable. Sous réserve d'un diagnostic et prise en charge précoce.

## Abstract (en)

Spontaneous neonatal gastric perforation is rare. We report the case of a newborn without any abnormality identified at delivery and whose mother had problem-free pregnancy. On the third day of life, he had a sudden onset of severe abdominal distension followed by bilious vomiting. Abdominal X-rays without treatment showed massive pneumoperitoneum and laparotomy showed a perforation at the level of the anterior gastric wall closed in a single layer closure. Postoperative course was uneventful. Spontaneous neonatal gastric perforation usually has a favorable outcome. Hence the importance of early diagnosis and patient management.

## Introduction

La perforation gastrique spontanée est rare chez le nouveau-né né à terme et représente10 à 16% des perforations gastro-intestinales néonatales. Plusieurs mécanismes sont avancés dans la genèse de la perforation avec des aspects anatomopathologiques particuliers. Le taux élevé de mortalité chez ces patients peut être améliorée par un diagnostic précoce et par une réanimation rapide qui nécessite une prise en charge chirurgicale. Nous rapportons ici un cas de perforation gastrique idiopathique survenus chez un nouveau-né à terme.

## Patient et observation

**Figure 1:**
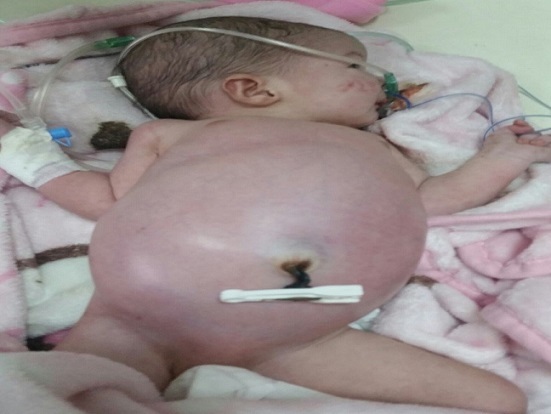
Distension abdominale importante

**Figure 2:**
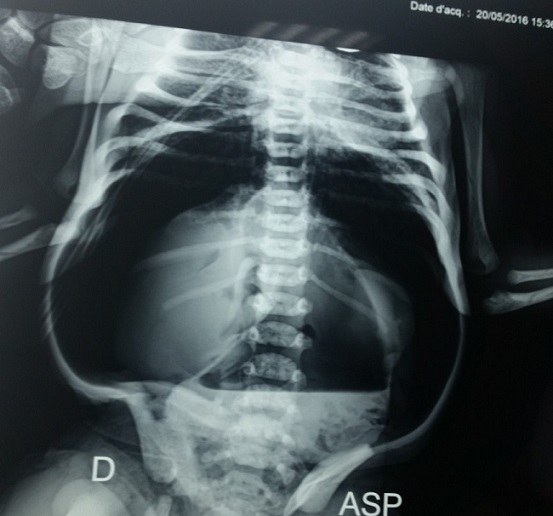
Aspect radiologique montrant un pneumopéritoine massif

Nouveau-né de sexe féminin né à terme, accouchement par voie basse avec présentation céphalique dans un hôpital régional, à la suite d'une grossesse mal suivie. Apgar à 10/10/10. A la naissance aucun manœuvre de réanimation, le poids était de 3700g, l'anamnèse infectieuse était négative. Au troisième jour de vie apparaissait une distension abdominale importante responsable de gêne respiratoire, compliquée 2 jours après, par des vomissements alimentaires puis bilieux et arrêt des matières et des gaz avec altération de l'état général. Il fut hospitalisé dans un hôpital régional pendant deux jours puis fut transféré dans notre service de réanimation néonatale à j 7 de vie pour prise en charge. A l'admission il était cyanosé, peu réactif et hypotonique. Sur le plan respiratoire, elle était tachypnéique à 60c/minute avec un score de Silverman à 5/10. La fréquence, cardiaque était de 150 c/minute et les pouls périphériques étaient perceptibles avec signes d'hypovolémie et plie de déshydratation. L'examen abdominal a trouvé une distension abdominale importante, (Figure 1), avec tympanisme diffus, sans masse palpable, sans aucun bruit hydro-aérique à l'auscultation abdominale Pas de malformations cliniquement décelables. Les aires ganglionnaires étaient libres. L'examen paraclinique réalisé a comporté : La radiographie de l'abdomen sans préparation qui montrait un pneumopéritoine massif, (Figure 2) évoquant une perforation d'un organe creux. Le nouveau-né était mis initialement sous oxygénothérapie par lunette, puis par sac à tête, une mise en place de sonde gastrique, une rééquilibration hydro électrolytique et une antibiothérapie associant céphalosporine de 3^ème^ génération, aminoside et métronidazole a été prescrite. Une ponction abdominale de décompression a été faite puis une laparotomie exploratrice était indiquée. Après correction des troubles hydro-électrolytiques, le malade est opéré par une incision transversale sus-ombilicale et ouverture du péritoine. L'exploration a trouvé une perforation antérieure au niveau de la grande courbure gastrique de 2cm, qui était suturée en un plan. Les suites opératoires étaient simples.

## Discussion

La perforation gastrique spontanée est rare chez le nouveau-né né à terme et représente 10 à 16% des perforations gastro-intestinales néonatales [1]. Depuis la première description de Siebold en 1825, plus de 300 cas ont été rapportés dans la littérature il est grave et peut mettre en jeu le pronostic vital. Elle intéresse 1/29000 naissances vivantes [2]. Dans notre pays, il est difficile de déterminer son incidence, en raison du manque des données épidémiologiques. La qualification de « spontanée » correspond à une entité propre et sont exclues les perforations gastriques associées à une obstruction distale [3 ,4]. Plusieurs facteurs de risque sont associés à l'affection : la prématurité, le faible poids de naissance, l'exsanguino-transfusion, la rupture prématurée des membranes, la toxémie gravidique, l'accouchement par le siège, le diabète maternel, le placenta prævia, l'infection amniotique ou encore la césarienne [5]. Par ailleurs plusieurs mécanismes sont avancés dans la genèse de la perforation avec des aspects anatomopathologiques particuliers selon l'étiologie. Ainsi, ont été rapportées les perforations d'origine congénitale par agénésie de la musculature gastrique occasionnant des lésions à type de déchirure linéaire au niveau de la grande courbure ; puis les perforations d'origine ischémique, d'origine mécanique, d'origine médicamenteuse ou encore d'origine fonctionnelle entraînant des perforations punctiformes de la paroi gastrique antérieure ou postérieure [3, 6]. Aucun de ces mécanismes ou facteurs de risque ne semble être en cause dans notre observation et l'origine reste indéterminée. L'âge habituel de survenue se situe entre deux et sept jours, avec un pic de fréquence au 3^ème^ jour [4]. Dans notre cas les premières manifestations cliniques sont apparues à j 3 de vie. Le tableau clinique de la perforation gastrique néonatale est assez caractéristique. En effet, lors des premiers jours alors que l'alimentation et l'émission de méconium sont normales, surviennent brutalement une distension abdominale, des vomissements et des troubles respiratoires qui évoluent en peu de temps vers une détresse respiratoire [4]. Dans notre cas, le principal symptôme était la distension abdominale puis la détresse respiratoire par compression diaphragmatique. La radiographie de l'abdomen sans préparation montre un pneumopéritoine massif caractéristique. Sur le plan thérapeutique, certains auteurs préconisent, avant la chirurgie, une ponction abdominale de décompression pour soulager le travail respiratoire. Le traitement chirurgical après rééquilibration hémodynamique consiste en une suture de la perforation qui peut par ailleurs être multiple, ce qui justifie une exploration minutieuse. Une toilette péritonéale avec une solution salée tiède et un assèchement de la cavité abdominale permettent, comme dans notre cas, d'éviter un drainage. L'évolution est la plupart du temps favorable pour les cas diagnostiqués et traités rapidement. La mortalité reste cependant élevée en cas de retard de la prise en charge, de grande prématurité ou de sepsis sévère associé [3]. Notre observation est comparable à celle de la littérature quant à sa description clinique et radiologique, ainsi que sur la localisation de la perforation au niveau de l'estomac. En revanche, sa particularité tient au fait qu'aucun des facteurs de risque décrit n'a été retrouvé : il ne s'agissait pas de prématurés et aucune manœuvre de réanimation à la naissance (ni aspiration gastrique, ni ventilation au masque) n'a été effectuée.

## Conclusion

La perforation gastrique chez le nouveau-né est une urgence chirurgicale rare mais grave qui met en jeu le pronostic vital. Les facteurs de risque classiques ne sont pas toujours retrouvés. L'évolution des perforations peut être favorable sous réserve d'un diagnostic et d'une prise en charge précoces.

## Conflits d’intérêts

Les auteurs ne déclarent aucun conflit d'intérêts.
